# No significant association between perioperative serum sodium and intestinal anastomotic leaks: a systematic review and meta-analysis

**DOI:** 10.1016/j.sipas.2026.100355

**Published:** 2026-07-15

**Authors:** Kaiser O'Sahil Sadiq, Eugenia Auxiliadora Marquez Castillo, Patricia Ruiz Cota, Andres Fontaine-Nicola, Samuel Eisenstein, Nicole Lopez

**Affiliations:** a Department of Surgery, Division of Colorectal Surgery, UC San Diego, San Diego, CA, USA; b Department of Surgery, Abington Jefferson Hospital, Abington, PA, USA; c Department of Surgery, Division of Minimally Invasive Surgery, UC San Diego, San Diego, CA, USA; d Department of Surgery, North Shore University Hospital, Zucker School of Medicine at Hofstra/Northwell, Manhasset, NY, USA

**Keywords:** Intestinal, Anastomosis, Anastomotic leak, Hyponatremia, Serum sodium

## Abstract

**Background:**

Anastomotic leakage (AL) rates have been reported as high as 40% with associated mortality approaching 27%. Non-osmotic activation of vasopressin may result in hyponatremia. It has been suggested that perioperative sodium changes may function as an inexpensive and widely available biomarker for early identification of AL.

**Methods:**

A systematic review and meta-analysis was performed, registered with PROSPERO (CRD42024522436). English language observational studies were eligible regardless of publication date. Methodological quality was evaluated using the QUADAS 2 tool. Quantitative synthesis was conducted using Meta Mar version 3.5.1.

**Results:**

Among 121 screened records, five studies fulfilled the inclusion criteria. One study demonstrated a high risk of bias related to patient selection, while the remaining studies were assessed as low risk across all evaluated domains. The combined cohort included 2034 patients, with AL occurring in 188 individuals (9%). Although all studies measured preoperative serum sodium, only three reported these values, and three studies assessed postoperative sodium levels. Pooled analysis showed no statistically significant differences in preoperative (SMD −0.45, *p* = 0.33) or postoperative sodium (SMD −3.61, *p* = 0.15) between patients with and without AL. In contrast, individual studies consistently demonstrated lower postoperative sodium levels among patients who developed AL that were statistically significant. Two studies identified postoperative hyponatremia or a postoperative decline in sodium as an independent predictor of AL. Reported sodium cutoffs ranging from 130 to 139.5 mEq/L yielded sensitivities between 23% and 70% and specificities between 72% and 93%.

**Conclusions:**

Current evidence does not demonstrate a statistically significant association between perioperative serum sodium levels and anastomotic leakage. While individual studies have suggested a potential relationship, the substantial heterogeneity and methodological limitations limit the reliability of these findings. Further well-designed prospective studies are required before serum sodium can be considered a clinically useful biomarker.

## Graphical abstract

**Figure:**
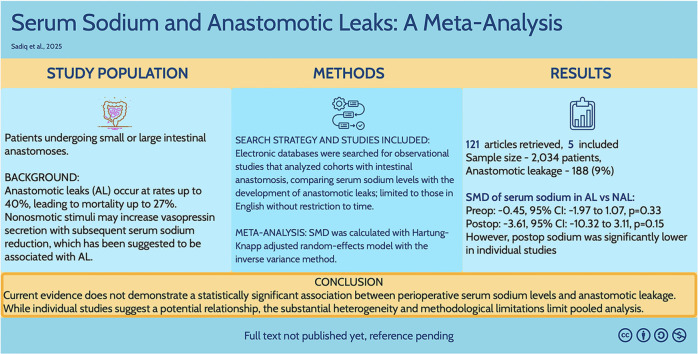

## Introduction

Over the course of almost three centuries, several discoveries and technical improvements have improved the safety and effectiveness of gastrointestinal anastomoses [1,2]. Yet despite these advances, anastomotic leaks (AL) continue to occur at rates between 1–40% [3,4], leading to mortality rates of 0.8–27% [5,6]. This represents an 8 to 10-fold increase compared to patients who do not develop AL [7], with significant added morbidity and financial costs [8]. The diagnosis of anastomotic leakage is primarily clinical. The Colon Leakage Score (CLS) has been designed to ascertain this risk preoperatively [9], and the Dutch Leakage (DULK) and modified DULK scores may be used for early identification in the postoperative period [10,11]. Of the 4 laboratory investigations used in the DULK score, only CRP was determined to be a highly predictive factor that was retained in the modification of this tool [11]. Procalcitonin has also been shown to be an effective serum marker for the early diagnosis of AL [[12], [13], [14]]. However, while these tests have high negative predictive values of 96–99%, their positive predictive value is only 21–34% [12,15,16]. Additionally, these tests are relatively expensive and may not be available for routine usage in developing countries and low-resource settings.

Hyponatremia is the most common electrolyte disorder encountered clinically [17]. Although the exact mechanism remains unclear, several non-osmotic stimuli may lead to increased antidiuretic hormone secretion and subsequent serum sodium reduction, [18,19] which has been seen to coincide with increased CRP [20]. Given its comparatively low cost and frequent usage as part of routine preoperative and postoperative evaluation, it has been hypothesized that serum sodium could have a role in the prediction or detection of anastomotic dehiscence; however, this remains unproven. To explore this hypothesis and assess the current state of the evidence, a systematic review of the published literature and subsequent meta-analysis was conducted.

## Methods

### Study aim

The primary outcomes for this study were to identify whether serum sodium levels of patients undergoing intestinal anastomoses are associated with anastomotic leakage, and similarly whether postoperative serum sodium levels are associated with anastomotic dehiscence. This serves to evaluate serum sodium as a preoperative risk factor and as a postoperative diagnostic aid.

### Study design

Conducted in accordance with PRISMA guidelines, [22] this systematic review and meta-analysis had its protocol prospectively registered on PROSPERO (CRD42024522436). The selected studies were screened and appraised for quality by 2 independent reviewers (K.O.S., E.A.M.C) who later extracted data from individual studies using a standardized table. When conflicts arose, the third author (P.R.C) decided the outcome.

### Search strategy

The literature search included EMBASE, the Cochrane Library, MEDLINE, PubMed, and PubMed Central, covering studies published through February 23, 2024. Cochrane Library was searched using (anastomo* OR anastomosis OR (anastomotic leak*) OR "anastomotic leakage") AND (hyponatremia) in the title, abstract, or keywords. Similarly, Pubmed, Pubmed Central, and Medline were searched using the following search strategy; (Hyponatremia OR "low sodium") AND (Anastomo* OR "Anastomotic leak*"). Embase was searched using Emtree terms. The following search string was employed: ’anastomosis leakage'/exp AND 'hyponatremia'/exp. Titles and abstracts were used to exclude irrelevant studies and those that did not correlate serum sodium levels with the development of anastomotic leaks. Manual searches of Google Scholar and reference lists from relevant articles were also conducted.

### Inclusion and exclusion criteria

Eligible studies analyzed cohorts undergoing small or large bowel anastomosis and evaluated serum sodium levels or hyponatremia rates in association with anastomotic leaks. Studies of other design types, such as case reports, in vitro studies, and animal studies, were omitted. Studies with anastomoses at other sites and articles in languages other than English were also excluded. Only studies involving human participants and published in English were considered, regardless of publication date, including studies published in the gray literature.

### Quality assessment

Studies were appraised for quality using the QUADAS-2 tool [23]. All studies were included in our analysis, irrespective of the results.

### Extracted data and variables

The data extracted included sample sizes, the mean age of patients, pathological diagnoses, sites of anastomoses, the proportion of anastomotic leaks, the definitions of hyponatremia used, serum sodium values, mortality as well as sensitivity and specificity when it was reported.

### Meta-Analysis

An exploratory quantitative synthesis was performed to supplement the systematic review. For studies that reported median values and ranges due to skewed distribution, the Method for Unknown Non-Normal Distributions (MLN) approach [24] was used to estimate mean and standard deviation. When the standard deviation was only depicted graphically, manual measurement of the image provided an estimate. Meta-Mar v3.5.1 was utilized for statistical analysis and visualization [25]. Standardized mean differences were calculated using a Hartung-Knapp adjusted random effects model with inverse variance method, and visualized as a forest plot [26]. Hedge’s g was used to estimate effect size, and heterogeneity was assessed using tau and I^2^.

## Results

**Fig. 1:**
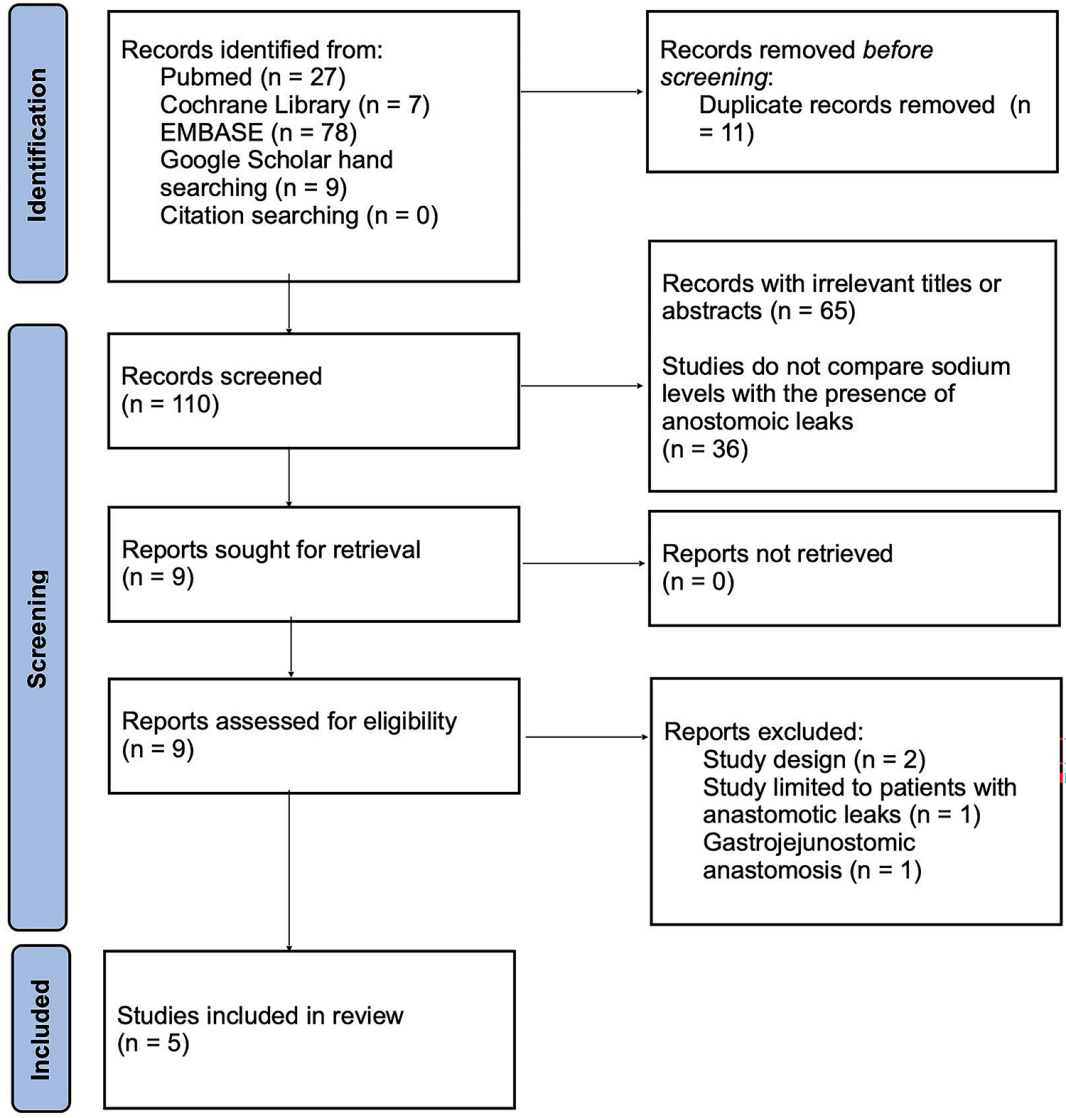
PRISMA (preferred reporting items for systematic reviews and meta-analyses) flowchart of study selection. Abbreviations: RCT, randomized controlled trial.

**Fig. 2:**
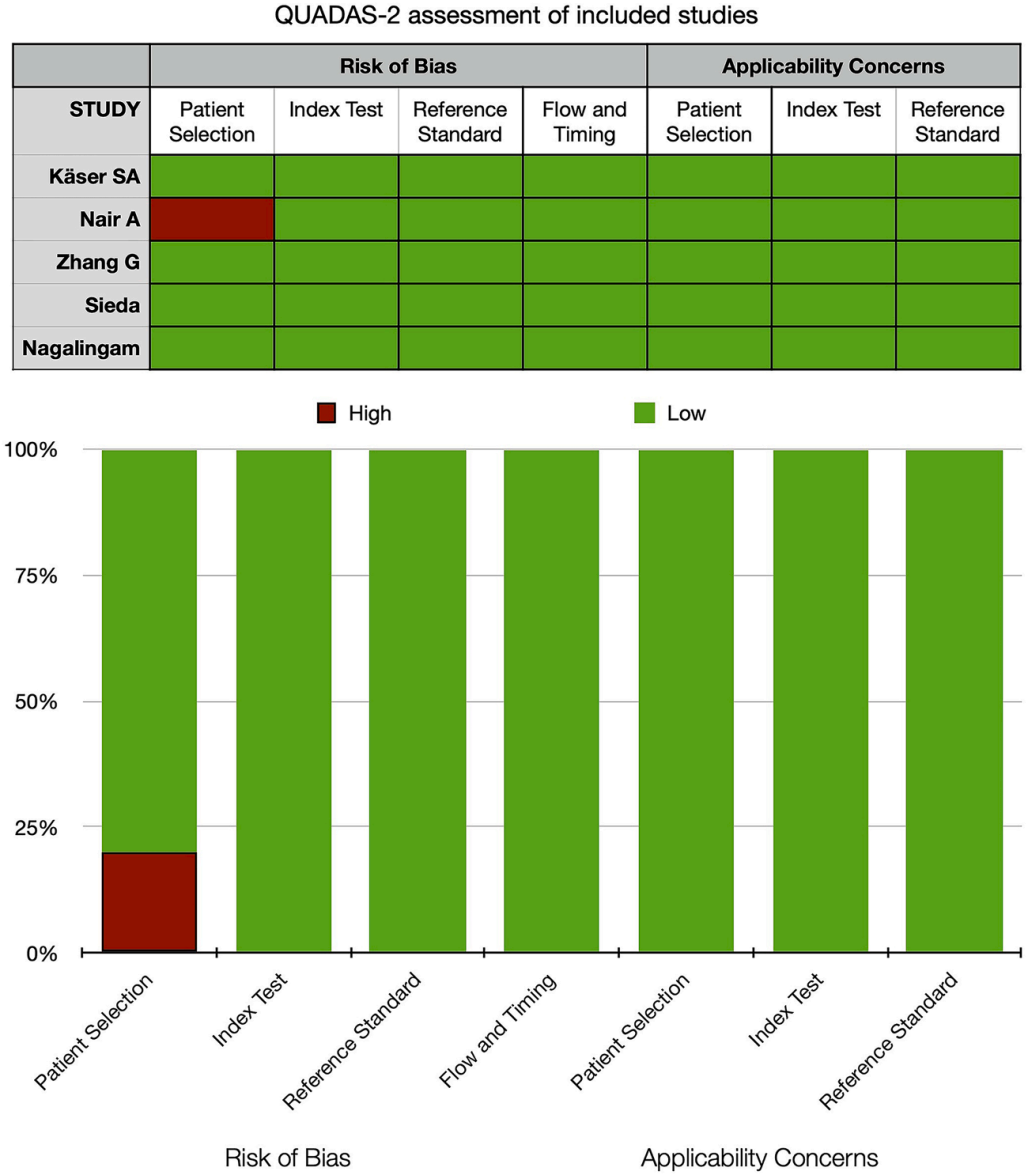
Quality appraisal using QUADAS-2 tool for included studies.

The search strategy resulted in 121 articles, 116 of which were excluded for various reasons, as shown in Fig. 1. Five articles [[27], [28], [29], [30], [31]] were assessed for quality, the results are displayed in Fig. 2. One study was deemed high risk due to selection bias related to the inclusion of predominantly high-risk surgical indications, [27] and the remaining were assessed to have a low risk of bias in all domains [[28], [29], [30], [31]]. In these studies, patient selection methods were deemed appropriate, with no evidence of inappropriate exclusions or selective enrollment. The index tests and reference standards were conducted and interpreted according to predefined protocols, with no significant concerns regarding diagnostic classification or outcome ascertainment. Furthermore, patient flow was considered appropriate, with all patients receiving the relevant index tests and reference standards and being included in the final analyses. Applicability concerns were likewise judged to be low, as the study populations, index tests, and reference standards were consistent with the objectives of the present review. All of these studies were included for review. The sample sizes of the included studies ranged between 70 and 1106 (median, 287), with a total sample size of 2034 patients. The data collected from each article is tabulated in Table 1.

**Table 1:** Study characteristics and measured outcomes.

First Author (Year)	Käser (2014)	Nagalingam (2021)	Nair (2006)	Sieda (2019)	Zhang (2020)
Study Design	Retrospective	Prospective	Prospective	Prospective	Retrospective
Study Location	Germany	India	India	Egypt	China
Sample size	1106	73	70	287	498
Average Age	62.2 (AL) 61.9 (NAL)	38.02 ± 16.26	41.5 (AL) 35 (NAL)	Divided into <65, >65. Cannot calculate. No significance	65.8 ± 10.7 (AL) 64.2 ± 12.7 (NAL)
% Male	67% (AL) 54% (NAL)	62%	69% (AL)	45%	70% (AL) 56% (NAL)
Pathology	Various	Various - perforation, gangrene, intestinal obstruction, leak	Various - Small bowel perforation, small bowel gangrene, intestinal obstruction	Various	Colorectal Cancer
Type of Anastomosis	Colonic and rectal Anastomosis	Enteroenteric and enterocolic anastomoses	Emergency enteroenteric and enterocolic anastomoses	Ileocolic, colocolic or enteroenteric	Colonic and rectal Anastomosis
Proportion of AL	81 (7.3%)	25 (34.2%)	26 (37.1%)	29 (10.1%)	27 (5.4%)
Diagnosis of AL	CT with contrast enema, conventional contrast enema, rectoscopy, intraoperative findings	NR	Clinical (bowel contents from wound or drain site), intraoperative findings	Clinical, diagnostic imaging or intraoperative findings	Clinical (feculant output from drain), CT or intraoperative findings
Hyponatremia Definition	<136	NR	NR	<130	<139.5
S. Na in AL	Preop: NR Postop: 138.8	Preop: 131.09 ± 6.32	Preop: 129 (122–144)	Preop: NR POD1: 130 (130–139) POD3: 124 (121–129)	Preop: 141.26 ± 2.61* Postop: 137.63 ± 4.29
S. Na in NAL	Preop: NR Postop: 140.5	Preop: 135.63 ± 7.51	Preop: 136 (123–158)	Preop: NR POD1: 134 (132–141) POD3: 134 (130–139)	Preop: 140.66 ± 2.90* Postop: 139.81 ± 3.41
Hyponatremia in AL	Preop: 2 (2%) Postop: 19 (23%)	NR	NR	24 (83%)	19 (70%)
Hyponatremia in NAL	Preop: 92 (9%) Postop: 69 (15%)	NR	NR	142 (55%)	130 (28%)
Mortality in AL	1 (1.2%)	13 (17.7%)	11 (15.7%)	Total: 17 (5.9%) No significant difference	NR
Mortality in NAL	NR	0%	1 (1.4%)		NR
Sensitivity	24%	NR	NR	23%	70.0%

Abbreviations: AL, Anastomotic Leak; NAL, No Anastomotic Leak; NR, Not reported; POD, Postoperative day; Postop, Postoperative; Preop, Preoperative; S. Na, Serum Sodium.

### Surgical indications

Most studies included various indications for surgery, such as perforation, gangrene, and obstruction. A single study focused exclusively on colorectal malignancy and excluded other indications for surgery [31]. This was the only study in this review that did not include anastomoses involving the small intestine.

### Proportion of anastomotic dehiscence

The methods employed to diagnose anastomotic leakage included intraoperative evaluation, imaging with contrast enemas and CT, and clinically by observing feculent discharge from drains or wounds. Across all articles, there were a total of 188 (9.2%) cases that developed anastomotic leakage, and this proportion ranged from a minimum of 5.4% [31] to a maximum of 37.1% [27].

### Serum sodium levels

All five studies recorded preoperative serum sodium levels, but the observed values were only reported in three studies [27,29,31]. Preoperative hyponatremia was only found to be statistically significant in two studies [27,28]. Three studies investigated postoperative serum sodium, [[29], [30], [31]] but these measurements were taken on several different days, and the values used for statistical analysis were decided by the investigators. Zhang used values closest to postoperative day (POD) 4, [31] Käser used values closest to POD 5 (Mean 5.2 days) in those without AL and values closest to the time of diagnosis for those that developed AL (Mean 8.4 days), [29] and Sieda measured sodium daily until POD7 but reported values POD1 and POD3 only [30]. For this meta-analysis, the sodium values Sieda et al. reported from POD3 were used, as this was closer in timing to the values used in the studies by Käser and Zhang, which is also within the timeframe where CRP has been shown to be of diagnostic value, as opposed to POD1. All three observed lower levels of sodium in cases of anastomotic leakage that were statistically significant [[29], [30], [31]].

### Mortality

Apart from Zhang, [31] all studies reported mortality rates [[27], [28], [29], [30]]. Käser reported only a single death in the AL group (1.2%), [29] Nair reported 11 deaths (15.7%) in the AL group and one death (1.4%) in the NAL group, [27] Nagalingam reported thirteen (52%) that exclusively occurred in the AL group, [28] and Sieda reported a total of 17 (5.9%) but did not specify the distribution among AL and NAL groups [30]. Nair found the difference in mortality to be statistically significant, [27] however, Sieda observed no statistically significant difference in their population [30].

### Sensitivity and specificity

Sensitivity and specificity were only evaluated in two of the studies [[29], [30], [31]]. Käser reported a sensitivity of 24% and a specificity of 93% when defining hyponatremia as any value lower than 136 mEq/L [29]. Using a higher threshold of 139.5 mEq/L, above the accepted lower limit of normal, Zhang observed the sensitivity to 70.4% but reduced specificity to 72.4% [31]. Similar to Käser, Sieda observed a sensitivity of 23% and a specificity of 93% when using a lower threshold of 130 mEq/L [30]. Since each of these studies used varying thresholds to define hyponatremia, meta-analysis of these results was not possible. The remaining studies did not report these measures.

### Meta-Analysis

**Fig. 3:**
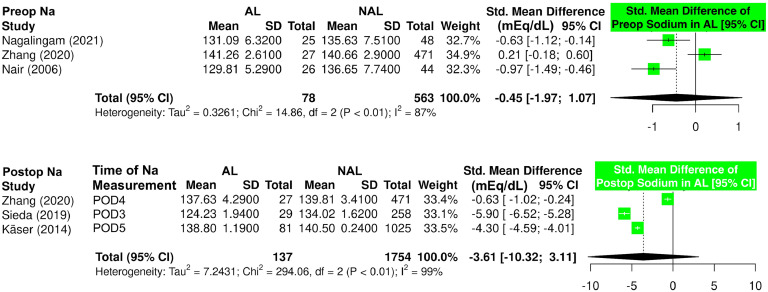
Forest plot of the comparison of serum sodium between those with and without anastomotic leakage. Notes: The random-effects meta-analysis model (Inverse Variance method) was used. Abbreviations: AL, Anastomotic Leakage; Cl, Confidence Interval; df, degrees of freedom; IV, inverse variance, Preop, preoperative; Postop, postoperative; Na, sodium; NAL, No Anastomotic Leakage; Std, Standard.

Meta-analysis of preoperative sodium levels from the three studies [27,28,31] where it was reported, resulted in a standardized mean difference (SMD) [95% CI] of −0.45 [−1.97, 1.07] (*p* = 0.33, I^2^ =86.5%, tau=0.57). Studies by Käser and Sieda could not be included in the meta-analysis due to the omission of the preoperative sodium levels [29,30]. Meta-analysis of postoperative sodium levels from the three studies [[29], [30], [31]] where it was reported, resulted in an SMD [95% CI] of −3.61 [−10.32, 3.11] (*p* = 0.15, I^2^=99.3%, tau=2.69). The forest plots for both of these are illustrated in Fig. 3.

## Discussion

AL remains a dreaded complication that may present with varying severity, ranging from asymptomatic to potentially fatal sepsis. AL develops at rates between 1–40%, [[3], [4]] leading to mortality of 0.8–27% [[5], [6]]. It has been estimated that AL increases mortality by a factor of 8–10 and doubles the length of hospital stay [7]. The different definitions of AL employed in the literature may explain the wide range of values observed [3,15,32]. The International Study Group of Rectal Cancer proposed a 3-tiered classification system based on the level of care needed, [33] which has been validated in another study [34]. No such rating systems were used in any of the studies included in this review. Several risk factors are known to increase the risk of AL but to the best of our knowledge, the only tool that formally stratifies this risk preoperatively is the CLS, which evaluates 11 factors, and high-risk anastomoses are those with a score over 11, corresponding to a 3% leak risk, [9] yet AL remains difficult to predict [35]. Innovative approaches to mitigating the risk of AL include real-time fluorescent imaging, which is being studied as an intraoperative tool to assess perianastomotic vascularity [36]. Owing to the late development of symptoms that are generally nonspecific, AL may not become clinically evident until POD 8–12.4 [37,38]. On average, AL is diagnosed on POD 6–12,38, [39] and earlier detection continues to be elusive [15,40]. Delayed diagnosis, particularly after POD 5, is associated with poorer outcomes [37]. With the wide adoption of Enhanced Recovery After Surgery (ERAS) protocols, AL may only become clinically apparent after discharge. Between radiation exposure, cost, and limited value in the absence of clinical suspicion, the routine use of postoperative imaging is not recommended, [29] and false negatives may lead to unnecessary surgeries [41]. Alterations in the perianastomotic microenvironment are believed to precede overt clinical manifestations, highlighting the potential utility of biomarkers as predictive tools [42].

Garcia-Granero found that CRP values under 147 and 135 mg/dl on days 3 and 5 have 99% and 98% negative predictive value, respectively, and shortened the time to diagnosis preceding clinical and radiological changes [12]. A 2014 meta-analysis showed CRP values on POD 3, 4, and 5 over 172 mg/dl, 124 mg/dl, and 144 mg/dl, respectively, to have a negative predictive value of 97% but a positive predictive value of only 21% to 23% [15]. While it has not been independently validated, [11] the DULK score incorporates CRP values as one of its criteria, has reportedly reduced mortality from 39 to 24%, and shortened the median time to diagnosis by 2.5 days [10]. While the DULK and modified DULK scores have high negative predictive values of 97%, their positive predictive value is only 16–17% [[10], [11]]. Procalcitonin is another marker that has shown promise, with the PREDICS trial showing a negative predictive value of 97% and 98% for values over 2.7 and 2.3 ng/dl on POD 3 and 5, respectively [16]. A 2018 meta-analysis found the greatest diagnostic performance for values over 1.26 ng/dl on POD4 [43].

While CRP and procalcitonin have been evaluated in substantially larger bodies of evidence, including multiple meta-analyses and prospective studies with defined diagnostic thresholds and validated scoring systems. The evidence base for serum sodium in this context is, by comparison, nascent and insufficient to draw meaningful comparisons regarding diagnostic performance. While postoperative serum sodium reduction has been proposed as a potentially cheaper and more routinely available laboratory value [21,44], the present meta-analysis did not demonstrate a significant association between postoperative serum sodium levels and AL, and no validated thresholds, sensitivity, specificity, or predictive values have been established for sodium in this setting. Therefore, the current evidence does not support positioning serum sodium as a comparable, complementary, or alternative biomarker to CRP or procalcitonin for the detection of AL. A previous systematic review [21] attempted to investigate the association between hyponatremia and anastomotic leakage. However, the previous authors included only one reference that evaluated anastomotic leaks. The authors of the present study sought to include articles that have since been published and attempted to retrieve relevant articles not included in the previous systematic review.

Hyponatremia occurs more commonly in postoperative patients as a result of the acute phase response, [45] and is thought to result from the up-regulation of antidiuretic hormone in response to various non-osmotic stimuli such as pain, nausea, vomiting, and volume depletion, which may be of greater magnitude in the event of AL and subsequent peritonitis [46,47]. This may be mediated through cytokines such as interleukin-1, interleukin-6, and tumor necrosis factor [48,49]. In contrast to Nair [27] and Nagalingam’s [28] finding of significant hyponatremia preceding AL, attributed to anastomotic edema, [28] there was no significant association between preoperative hyponatremia and AL in larger studies by Käser and Sieda [[29], [30]]. Our meta-analysis showed no significant association either. Since the etiology of AL is multifactorial, it is possible that the inclusion of inflammatory surgical indications such as gangrene, obstruction, and perforation may have influenced the findings of Nair and Nagalingam, contributing to the development of hyponatremia and increasing the risk of AL independently. Therefore, the role of preoperative hyponatremia as a risk factor for AL is debatable. While our meta-analysis failed to find a significant association between postoperative serum sodium and AL, this was not the case in each of the studies where it was assessed [[29], [30], [31]]. Through logistic regression analysis, lower postoperative serum sodium was found to be an independent predictive factor by both Zhang (odds ratio, 1.176, *p* = 0.006) [31] and Käser (95% CI, 0.154 to −0.037, *p* = 0.001) [29]. Additionally, a drop in sodium levels was associated with AL in studies by Käser and Zhang [[29], [31]]. This discrepancy may reflect the heterogeneity or selection bias. The datasets generated were not available for more granular analysis of this discrepancy.

This present systematic review and meta-analysis is limited by the low number of publications that assessed sodium levels in relation to anastomotic leakage in heterogeneous patient populations. Additionally, the methodology varied widely between studies in terms of indications for surgery, as well as sites of anastomoses. Known risk factors for AL include age, male gender, level of anastomosis, obstruction and peritonitis, intraoperative challenges, and blood transfusions [9,50]. The investigations included in this analysis were conducted in different geographic locations, and differences in healthcare practices may have affected the findings and conclusions. Four out of the five studies included various underlying pathological diagnoses, diagnostic methods were not clearly defined, thresholds for hyponatremia and timing of laboratory evaluation of the sample varied across the included studies, representing further causes of heterogeneity. This heterogeneity may have also contributed to the nonsignificant results observed from the present meta-analysis. Given this high heterogeneity, the pooled effect estimate should not be interpreted as a reliable summary of the underlying evidence. Another key limitation is the inconsistency in the timing of sodium measurements across the included studies. In some studies, sodium was measured on fixed postoperative days, whereas in others it was recorded near the time of anastomotic leak diagnosis. This discrepancy introduces a critical issue of temporality: when sodium is measured at or around the time of clinical diagnosis, any observed reduction may represent a physiologic consequence of an already evolving leak rather than a preceding signal. As such, the current body of evidence does not permit a clear distinction between sodium as a predictor of future anastomotic leak, a marker for early detection of a subclinical leak, or simply a correlate of established disease. Future studies should explicitly define the temporal relationship between sodium measurement and leak onset, and the framing of results should reflect whether the data support prediction, early detection, or association alone. The absence of a uniform definition of hyponatremia across the included studies further limits our findings. The thresholds used to classify patients as hyponatremic ranged from 130 mEq/L to 139.5 mEq/L, which includes values considered within the normal reference range by standard clinical definitions. Due to the design of the included literature being confined to observation studies, the lack of blinding, control groups, and inconsistent adjustment for confounders, including age, comorbidities, surgical technique, and intraoperative conditions, may have led to biases in the workup and resulting data. The observed associations may not be independent. Since the workup for possible AL is initiated based on subjective clinical assessment, it is possible that an increased incidence of AL was detected in patients with hyponatremia as a result of increased clinical suspicion due to this abnormal laboratory investigation. The included studies did not make note of sodium repletion that may have occurred through the administration of intravenous fluids or other medications that may have influenced serum sodium levels, except for Zhang, who noted that sodium supplementation was not regulated [31]. Serum sodium reduction does not exclusively occur with the administration of hypotonic fluids, but may also occur with isotonic fluids as well [[18], [19]]. Asymptomatic and slowly developing AL may have eluded detection prior to discharge, the inclusion of which may have led to different results. Patients should ideally be followed for a minimum of 30 days to detect late-onset AL.

Although the individual studies consistently reported an association between postoperative sodium reduction and anastomotic leakage, the methodological limitations and risk of residual confounding preclude definitive conclusions regarding causality or diagnostic performance. Consequently, the current evidence is insufficient to support serum sodium as a reliable standalone diagnostic or predictive biomarker for anastomotic leakage. Rather, these findings should be considered hypothesis-generating and underscore the need for well-designed prospective studies using standardized sodium measurement protocols and consistent outcome definitions to better define the potential role of postoperative serum sodium changes in the early detection of anastomotic leakage.

Based on these findings, larger, prospective double blind observational studies are needed to definitively clarify the association between postoperative serum sodium levels and anastomotic leak, as well as to assess the diagnostic value of perioperative sodium trajectories. Evaluating sodium levels beyond 5 days may not be feasible for those with an uneventful postoperative period; however, to reduce bias, values should be recorded with consistent timing and compared with similar values. Comparative trials evaluating CRP and serum sodium would be necessary before any conclusions can be drawn regarding sodium as an alternative biomarker, a role that is not supported by the present findings. For robust results, we suggest that logistic regression be performed during statistical analysis to account for potential confounding factors and evaluate serum sodium as an independent variable.

## Conclusion

Serum sodium levels measured preoperatively do not appear to correlate with an increased risk of anastomotic dehiscence. Meta-analysis showed no significant association between postoperative serum sodium reduction and AL, despite being seen in three individual studies. These findings should be regarded as hypothesis-generating only and do not support the clinical use of postoperative sodium as a diagnostic or predictive marker for anastomotic leak at this time. If postoperative sodium is a viable serum marker to help identify anastomotic leaks, further investigations may be required to establish this relationship. Even when within the normal range, a postoperative decrease in serum sodium levels compared to preoperative levels merits further study as a potential diagnostic marker; however, this remains speculative and requires validation before any clinical application can be considered.

## Funding

No financial support or funding of any kind was received to conduct, author, or publish this article.

## Disclaimer

Any opinions expressed in this paper are solely those held by the authors and are not representative of either the institution or the publisher.

## Disclosure

S.E. is a consultant for Takeda. There are no other conflicts of interest to disclose. No financial support or funding of any kind was received to conduct, author, or publish this article.

## Meeting presentations

Sponsoring Organization: American Society of Colon and Rectal Surgeons (ASCRS).

Location: San Diego, CA.

Date: May 11, 2025.

Sponsoring Organization: The Society of Asian Academic Surgeons (SAAS).

Location: Huntington Beach, CA.

Date: Sep 4–5, 2025.

Sponsoring Organization: European Society of Coloproctology (ESCP).

Location: Paris, France.

Date: Sep 10–12, 2025.

## CRediT authorship contribution statement

**Kaiser O'Sahil Sadiq:** Writing – original draft, Visualization, Methodology, Investigation, Formal analysis, Data curation, Conceptualization. **Eugenia Auxiliadora Marquez Castillo:** Writing – review & editing, Formal analysis, Data curation. **Patricia Ruiz Cota:** Writing – review & editing, Formal analysis, Data curation. **Andres Fontaine-Nicola:** Writing – review & editing, Formal analysis. **Samuel Eisenstein:** Writing – review & editing, Validation, Supervision. **Nicole Lopez:** Writing – review & editing, Visualization, Supervision.

## Declaration of competing interest

I, Kaiser Sadiq, hereby declare that Samuel Eisenstein is a consultant for Takeda. Aside from this, we have no other conflicts of interest to disclose. I confirm that neither I nor any of the remaining co-authors have personal, financial, or professional relationships that could be perceived as influencing our objectivity or integrity in this matter. I understand the importance of transparency and will promptly inform relevant parties should any potential conflicts arise in the future.
